# Case Report: Image-enhanced endoscopic characteristics of gastric amyloidosis with narrow-band imaging comparison

**DOI:** 10.3389/fmed.2026.1683757

**Published:** 2026-01-29

**Authors:** Xueman Wang, Bo Lian, Quan Luo, Xintong Jiang, Jian Dong, Tiannu Ding, Qiaoying Zhang, Yan Tang, Lifeng Hu

**Affiliations:** 1 Endoscopy Center, Shaoxing People's Hospital (The First Affiliated Hospital, Shaoxing University), Shaoxing, Zhejiang, China; 2 Forensic Center, Shaoxing University, Shaoxing, Zhejiang, China

**Keywords:** Congo red staining, endoscopy, gastric erythema, gastrointestinal amyloidosis, *Helicobacter pylori*, light-chain amyloidosis (AL)

## Abstract

**Background:**

Gastrointestinal (GI) amyloidosis is a rare disorder with nonspecific clinical and endoscopic features, often leading to misdiagnosis. Timely recognition is crucial to prevent diagnostic delays and systemic complications.

**Case Presentation:**

A 64-year-old male initially presented with upper respiratory symptoms, and chest computed tomography (CT) performed for pneumonia evaluation incidentally revealed esophageal wall thickening. The first endoscopy showed diffuse redness with loss of the regular arrangement of collecting venules in the gastric body, and the findings were initially interpreted as Helicobacter pylori–associated gastritis. However, both gastric histopathology and the ¹³C urea breath test were negative, and no clear cause of erythema was identified. The patient’s symptoms resolved after taking anti-inflammatory medication, and he did not return for follow-up as instructed. One year later, the patient developed proteinuria and bilateral lower extremity edema and was diagnosed with systemic light-chain (AL) amyloidosis involving the kidneys and heart. Repeat endoscopy revealed disc- and linear-shaped gastric erythema with a submucosal tumor (SMT)-like protrusion. Congo red staining with polarized light confirmed amyloid deposition in the gastric biopsy. In addition, we compared the endoscopic features of gastric erythema from three different etiologies. On narrow-band imaging (NBI), amyloid-related erythema showed a grayish-green signal with a more layered, deeper-appearing distribution, whereas vonoprazan-associated and Helicobacter pylori–associated erythema appeared more superficial with limited layering. Because absolute color intensity is not consistently comparable across panels, this observation should be considered hypothesis-generating rather than diagnostic.

**Conclusion:**

This case highlights the diverse and potentially misleading endoscopic manifestations of GI amyloidosis. NBI may aid in visualizing the distribution pattern and apparent depth of amyloid-related mucosal signals and serve as a supportive diagnostic tool; however, histological confirmation via Congo red staining remains the gold standard. The combination of image-enhanced endoscopy, targeted biopsies, and multidisciplinary evaluation may facilitate earlier recognition and more comprehensive management of patients with atypical gastric erythema.

## Introduction

Gastrointestinal amyloidosis (GIA) is a rare and potentially underdiagnosed condition characterized by the extracellular deposition of misfolded amyloid proteins in the gastrointestinal (GI) tract (1). Although amyloidosis can affect any part of the GI tract, the small bowel and stomach are most commonly involved (2, 3). Clinical manifestations include bowel dysmotility, weight loss, gastrointestinal bleeding, ulceration, perforation, and malabsorption (4). However, these symptoms are often non-specific, leading to diagnostic delays and misdiagnosis as other gastrointestinal disorders (5). The heterogeneous clinical presentation and the absence of distinct biomarkers or imaging techniques for early detection further complicate the diagnosis of GIA, often resulting in missed opportunities for early intervention (6, 7).

Endoscopic findings in GIA are highly heterogeneous and closely correlated with the deposition pattern of amyloid fibrils. In light-chain (AL) amyloidosis, amyloid typically accumulates in the muscularis mucosae, submucosa, and muscularis propria, leading to erythema, erosions, ulcers, polypoid lesions, and submucosal tumor (SMT)-like protrusions (8–10). Gastric involvement may manifest with fine granularity, mucosal friability, and erosions—findings that closely resemble *Helicobacter pylori*-associated gastritis. However, the varied and often subtle endoscopic appearances contribute to frequent misdiagnosis, as these presentations are often mistaken for more common gastrointestinal conditions. Although gastric biopsy with Congo red staining remains the diagnostic gold standard, it is typically performed only after systemic manifestations develop, underscoring the need for earlier recognition at the mucosal level (11).

Recent advances in image-enhanced endoscopy (IEE), particularly narrow-band imaging (NBI), may offer new opportunities for improving early detection (12). By enhancing mucosal microstructures and vascular patterns using optical wavelength filtration, NBI allows for subtle mucosal changes to be visualized with greater clarity (13, 14). Such optical differences may help differentiate amyloid-related mucosal changes from other etiologies of gastric erythema.

In this report, we present a patient with gastric amyloidosis initially misdiagnosed as *Helicobacter pylori*-associated gastritis, later confirmed to have systemic light-chain (AL) amyloidosis. Through comparison with vonoprazan-associated *and Helicobacter pylori*-associated gastric erythema, the more consistent and convincing NBI distinction was the distribution pattern and apparent depth (layering) of the grayish-green signal, rather than differences in absolute green intensity. This observation suggests that NBI may aid in differentiating GIA from other erythematous conditions with similar white-light appearances.

## Case presentation

**Figure 1:**
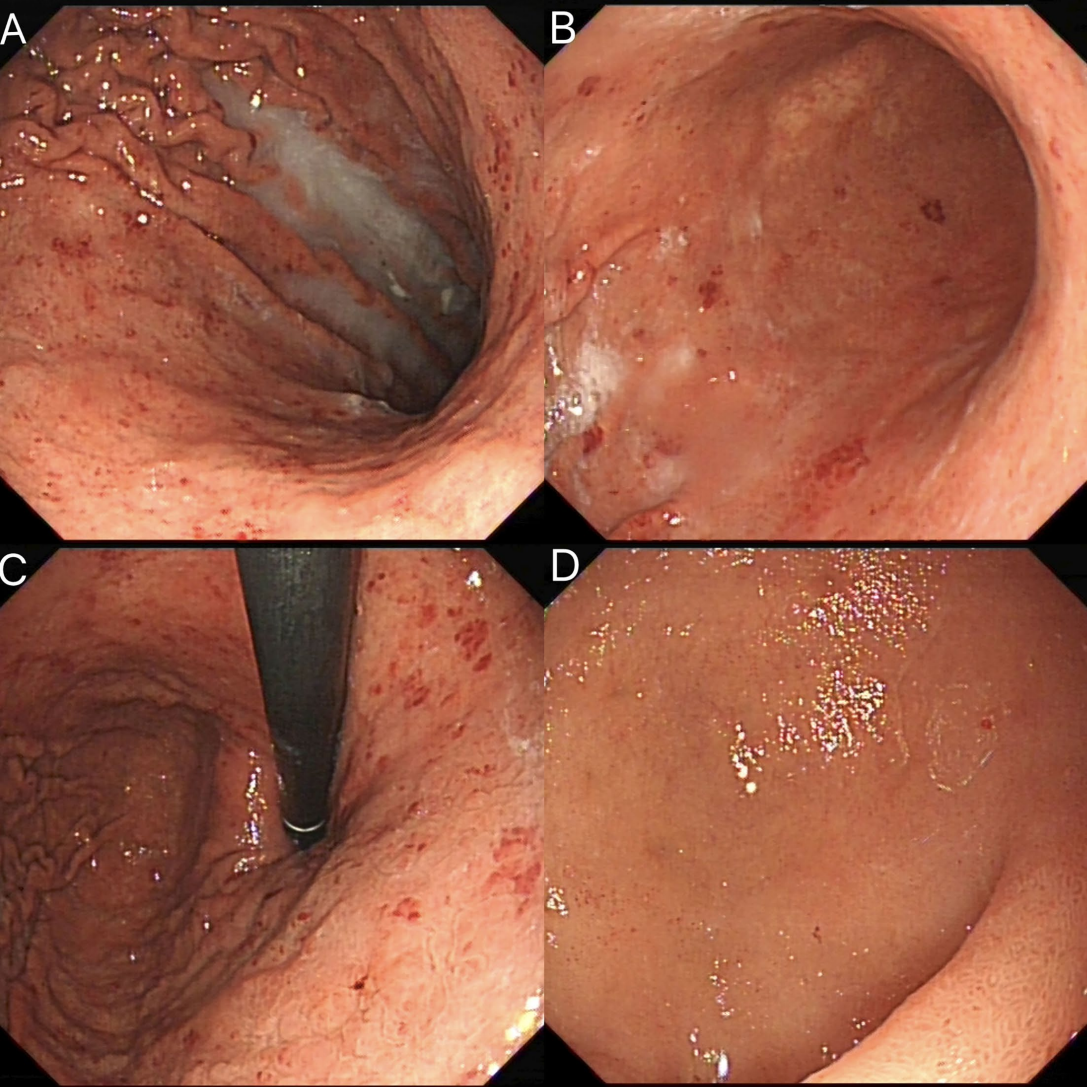
Initial endoscopic findings: **(A)** diffuse redness with scattered red erythema; **(B)** diffuse redness with sticky mucus in corpus; **(C)** RAC visible in body; and **(D)** duodenal erythematous spots.

A 64-year-old man presented to the ear, nose, and throat (ENT) department on 17 April 2023, with a several-month history of hoarseness, sore throat, and coughing with sputum production. The patient had a history of hypertension for 20 years and was on long-term antihypertensive therapy with 80 mg of valsartan and 50 mg of losartan. He had a 50-pack-year smoking history (10 cigarettes per day) and had never attempted to quit. There was no notable family history of disease. Physical examination revealed normal vital signs, chronic congestion of the pharyngeal mucosa, and mild bilateral tonsillar enlargement (Grade 1) without erythema or suppuration. Laboratory tests showed a hemoglobin level of 111 g/L and C-reactive protein (CRP) of 53.56 mg/L. Chest CT revealed a subpleural lesion in the left lung, suggestive of chronic inflammation, with follow-up recommended after treatment. Mild thickening and blurring of the esophageal wall prompted further evaluation. Flexible laryngoscopy revealed mild lymphoid follicular hyperplasia in the posterior pharyngeal wall, without swelling or erythema of the epiglottis, and slight edema and polypoid growth in the anterior parts of both vocal cords. The clinician suspected an upper respiratory infection and prescribed oral cefuroxime axetil for treatment. Due to the thickening of the esophageal wall seen on CT imaging, tumor markers for esophageal cancer were tested, revealing an elevated squamous cell carcinoma (SCC) antigen level of 1.84 ng/mL, which prompted an immediate upper gastrointestinal endoscopy. The endoscopic examination revealed diffuse redness with scattered red erythema and sticky mucus in the gastric lumen. Notably, the regular arrangement of collecting venules (RAC) in the gastric angle was absent (Figures 1A–C), and scattered erythematous spots were also observed in the duodenum (Figure 1D). Based on these findings, the initial diagnosis was *Helicobacter pylori*-associated gastritis. The endoscopist performed a routine biopsy from the gastric antrum, and the pathological examination showed mild chronic superficial gastritis. Both *Helicobacter pylori* pathology testing and the 13C urea breath test were negative. Congo red staining was not performed on the initial biopsy specimens. After receiving anti-inflammatory medications, the patient’s upper respiratory tract infection symptoms improved, and he did not return for further follow-up.

On 14 June 2024, the patient presented to nephrology with bilateral lower limb edema and no other significant symptoms. Physical examination revealed bilateral pitting edema in both lower extremities. Laboratory tests revealed anemia, with a hemoglobin level of 115 g/L (reference: 130–175 g/L). Urinalysis showed 2 + proteinuria, and 24-h urine protein quantification reached 4106.17 mg/24 h (reference: <150 mg/24 h). Serum albumin was decreased at 27.5 g/L (reference: 40–55 g/L), and creatinine was elevated at 118.8 μmol/L (reference: 59–104 μmol/L), with an estimated glomerular filtration rate (eGFR) of 55.8 mL/min (reference: >90 mL/min), indicating renal impairment. The erythrocyte sedimentation rate (ESR) was markedly elevated at 102 mm/h (reference: 0–15 mm/h), and C-reactive protein (CRP) was 29.56 mg/L (reference: 0–6 mg/L), suggesting active inflammation. Urinary light-chain analysis revealed elevated *λ*-light chains at 26.4 mg/L (reference: <3.9 mg/L) and *κ*-light chains at 21.8 mg/L (reference: <7.1 mg/L). Immunofixation electrophoresis revealed monoclonal immunoglobulin A-*λ* (IgA-λ) in both serum and urine. Renal ultrasound showed no abnormalities. Cardiac ultrasound revealed left atrial enlargement, degenerative changes in the aortic valve, and mild regurgitation of the aortic, mitral, tricuspid, and pulmonary valves. Abdominal ultrasound showed fine liver echogenicity with dilation of the common bile duct. On 19 June 2024, the patient underwent a kidney biopsy, which confirmed the presence of amyloid deposits in the kidneys (AL type). On 23 June, the patient was referred to the Peking Union Medical College Hospital (PUMCH) for further evaluation, where cardiac involvement was confirmed through myocardial perfusion delayed imaging dynamic MRI, which revealed left atrial enlargement and diffuse thickening of the interventricular septum and left ventricular wall. The diagnosis of primary light-chain amyloidosis (PAL) was made. Over the subsequent year, the patient underwent multiple cycles of DVd chemotherapy (daratumumab, bortezomib, and dexamethasone), with a limited therapeutic response. He is currently receiving weekly treatment with 60 mg of telitacicept.

**Figure 2:**
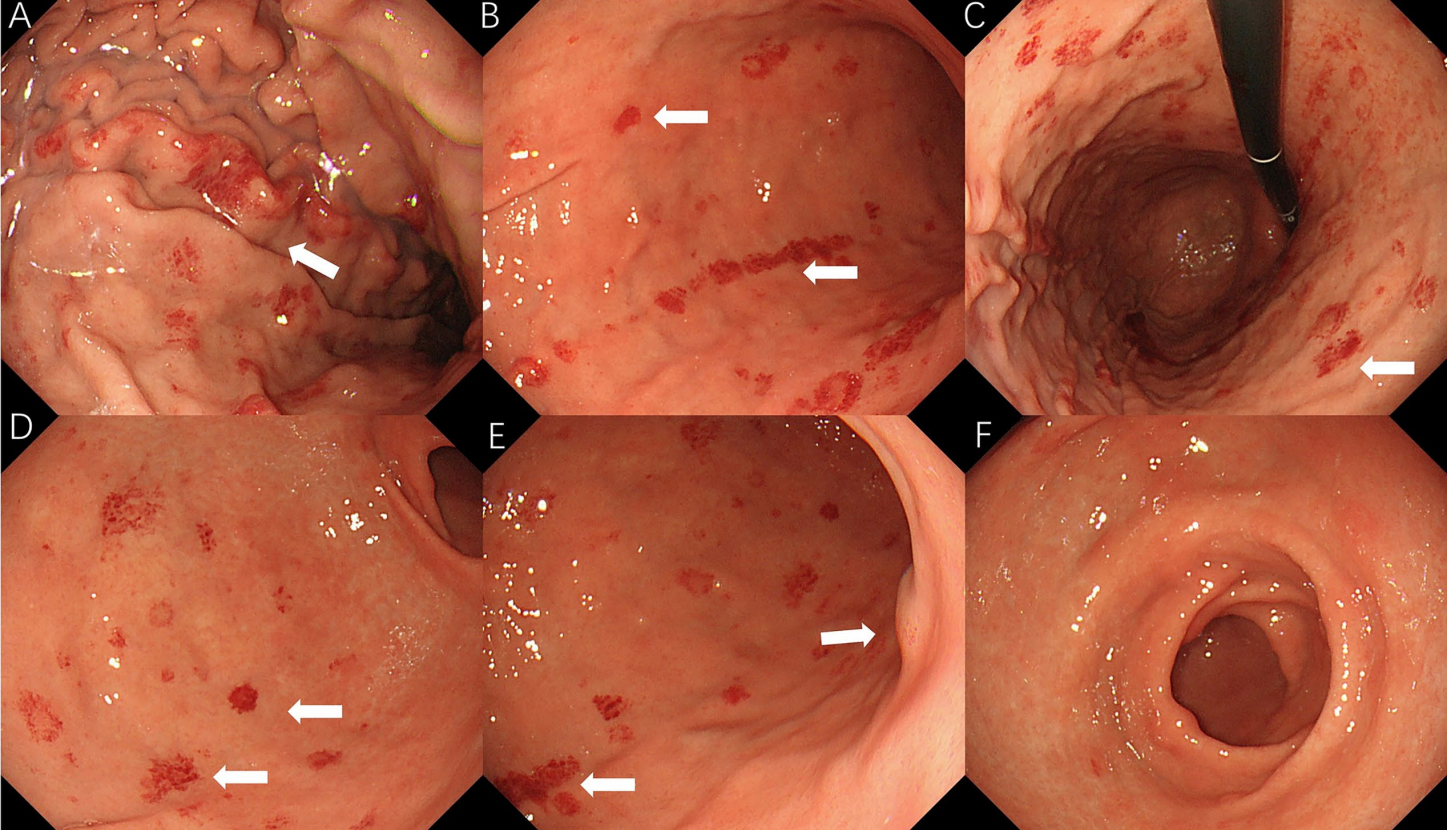
2025 endoscopic findings: **(A–D)** disc/linear gastric erythema; **(E)** SMT-like protrusion; and **(F)** antrum without erythema.

**Figure 3:**
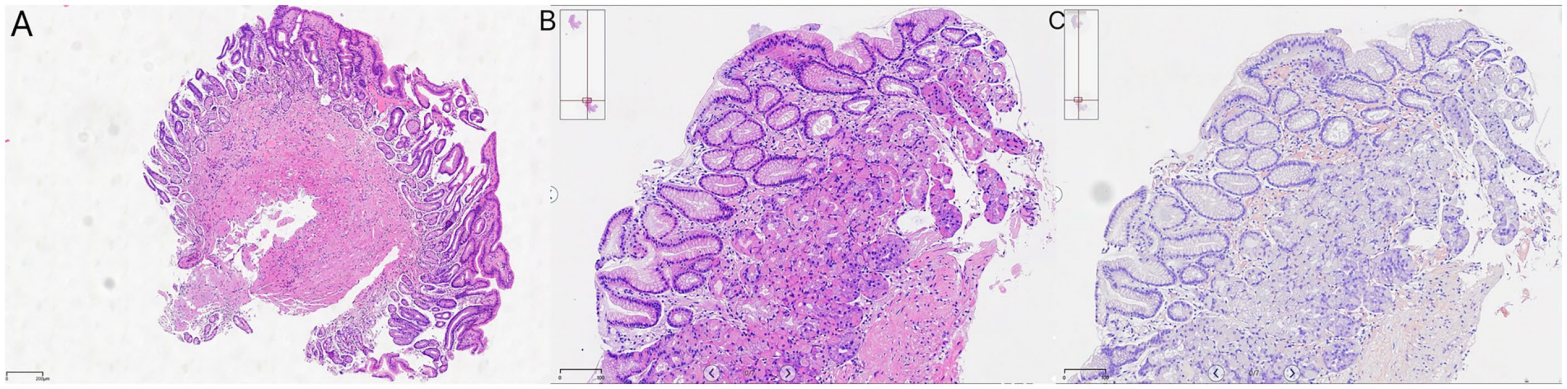
Histopathology: **(A)** 2023 antral biopsy (hematoxylin & eosin [H&E], ×40 magnification); **(B)** 2025 gastric body (H&E, ×40 magnification); and **(C)** Congo red staining with birefringence under polarized light, ×40 magnification.

On 10 April 2025, the patient underwent repeat endoscopy due to new-onset gastroesophageal reflux symptoms. The endoscopy revealed disc- and linear-shaped erythema in the gastric body with abnormally bright reddish coloration (Figures 2A–D). A submucosal tumor (SMT)-like protrusion was observed in the posterior wall of the lower gastric body (Figure 2E). In contrast, the gastric antrum showed no visible erythema (Figure 2F). Biopsy samples from these lesions showed eosinophilic deposits in the lamina propria (Figure 3B). Congo red staining was weakly positive, and apple-green birefringence under polarized light confirmed amyloid deposition (Figure 3C). Retrospective review of the 2023 biopsy revealed previously overlooked eosinophilic deposits (Figure 3A).

**Figure 4:**
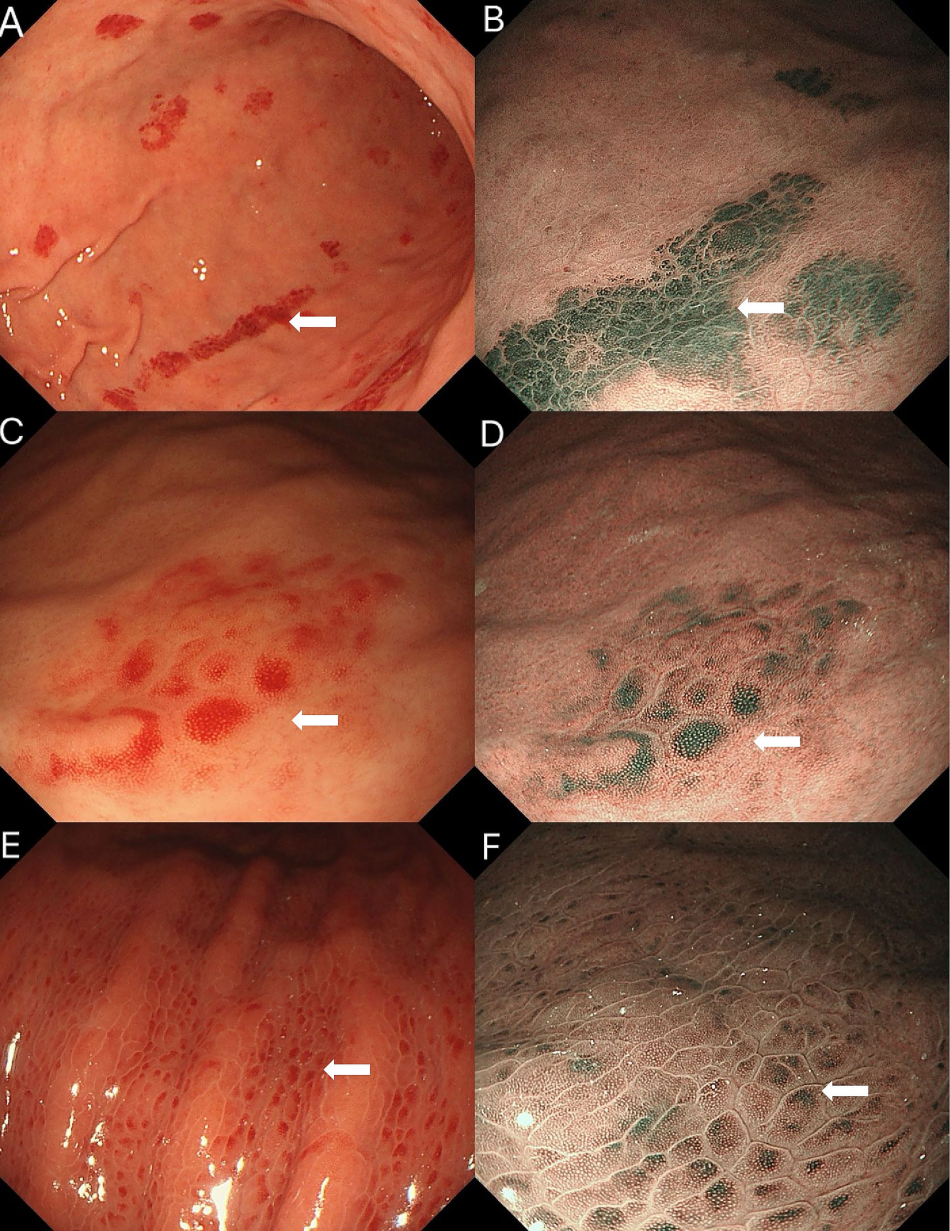
Comparison of gastric erythema of three etiologies. **(A, B)** Amyloid-related erythema: **(A)** white-light imaging shows bright discoid and linear erythema; **(B)** on NBI, the lesion shows a grayish-green signal with a layered, deeper-appearing distribution. **(C,D)** Vonoprazan-induced erythema: **(C)** white-light imaging shows spotty redness on the greater curvature of the gastric body; **(D)** on NBI, the lesion shows a relatively superficial pattern with minimal apparent layering. **(E,F)** *Helicobacter pylori*-associated gastric erythema: **(E)** White-light imaging shows densely distributed, uniform red spots; **(F)** on NBI, the erythema shows a patchy green signal with a reddish-brown halo in the surrounding mucosa and indistinct borders, with little apparent layering (more superficial-appearing).

We further compared the endoscopic features of gastric erythema across three etiologies. On NBI, amyloid-related erythema showed a grayish-green signal with a more layered, deeper-appearing distribution, whereas the comparator lesions appeared more superficial with limited apparent layering (Figure 4). Additionally, amyloid-related erythema showed relatively well-defined borders with mild mucosal thickening.

## Discussion

### Challenges in diagnosing gastrointestinal amyloidosis (GIA)

Gastrointestinal amyloidosis (GIA) is a rare and underdiagnosed disorder characterized by the extracellular deposition of amyloid proteins in the gastrointestinal (GI) tract (15). One of the main challenges in diagnosing GIA is its non-specific clinical and endoscopic features, which often overlap with more common GI disorders, leading to diagnostic delays (15). In our patient, the initial endoscopic finding of diffuse gastric erythema was interpreted as *Helicobacter pylori*-associated gastritis—a common and often correct assumption. However, histological examination and the urea breath test ruled out *H. pylori* infection, and the patient had no history of portal hypertension or non-steroidal anti-inflammatory drug (NSAID) use; no other clear cause for the erythema was identified. This case highlights a common pitfall in clinical practice—the default to common diagnoses when faced with non-specific endoscopic findings, thereby overlooking rare conditions such as GIA. This “missed opportunity” underscores the importance of considering a broad differential diagnosis when encountering unexplained, persistent gastric erythema. Given the potential for GIA to mimic other gastrointestinal conditions (16, 17), it is essential for clinicians to remain vigilant and consider rare etiologies. Early diagnostic workups should include more comprehensive biopsy strategies, such as targeted biopsies with Congo red staining, to rule out GIA. By doing so, it may be possible to catch cases of amyloidosis before systemic involvement occurs, thereby improving patient outcomes and reducing diagnostic delays.

### Diagnostic challenges and the role of targeted biopsies

Upon retrospective analysis of the patient’s gastric biopsy from 2023, we identified small eosinophilic deposits in the muscularis mucosae that had initially been overlooked. This misdiagnosis can be attributed to both clinical sampling and pathological interpretation limitations (18). The biopsy was not obtained from the most prominent erythematous areas observed during endoscopy, where amyloid deposits were likely concentrated. Additionally, the pathologist did not suspect amyloidosis at the time, and the small eosinophilic deposits were misinterpreted as fibrous tissue or non-specific exudates. This case highlights the need for improved collaboration between clinicians and pathologists. Clear clinical suspicion of rare diseases such as amyloidosis should be communicated to the pathology team to trigger appropriate diagnostic staining, such as Congo red (19). Furthermore, clinicians should consider deeper or targeted biopsies for patients with persistent, unexplained gastric lesions that do not respond to conventional treatments, especially when common causes have been ruled out. In cases of unexplained gastric erythema, especially when accompanied by other systemic signs, early recognition and biopsy can be pivotal in identifying rare conditions such as GIA.

### Potential of image-enhanced endoscopy in early diagnosis

Image-enhanced endoscopy (IEE), particularly narrow-band imaging (NBI), has the potential to aid in the early diagnosis of gastrointestinal amyloidosis (20–22). In our case, the more consistent and convincing NBI distinction lay in the distribution pattern and apparent depth (layering) of the grayish-green signal, rather than in absolute green intensity. This finding is biologically plausible, as extracellular amyloid deposits in deeper layers of the gastric mucosa, such as the submucosa and muscularis mucosae, may alter light absorption and scattering properties. In contrast, vonoprazan-induced erythema mainly results from superficial mucosal congestion (23, 24), while *Helicobacter pylori*-related erythema is characterized by neutrophil infiltration in the gastric epithelium (25). These NBI characteristics may serve as a valuable tool for differentiating amyloidosis-related mucosal changes from other causes of gastric erythema (12). However, these findings should be considered speculative and hypothesis-generating. Further studies with larger patient cohorts are needed to validate the utility of NBI as a diagnostic adjunct for GIA. Given the heterogeneity of gastric amyloidosis and overlapping endoscopic features with other forms of gastritis, NBI should be considered an adjunctive tool rather than a definitive diagnostic marker. Future studies should focus on developing standardized NBI criteria for GIA, which could complement histopathological findings and improve diagnostic accuracy.

### Systemic implications and the need for a multidisciplinary approach

The systemic nature of amyloidosis highlights the importance of a multidisciplinary approach to diagnosis and management. In our case, gastrointestinal symptoms appeared approximately 1 year before the development of renal and cardiac involvement, suggesting that early gastrointestinal manifestations may serve as key indicators of systemic amyloidosis. Early recognition of gastric amyloidosis could have led to a quicker referral to hematology, facilitating earlier systemic evaluation and treatment initiation. In patients with suspected systemic amyloidosis, a thorough evaluation of the gastrointestinal tract is essential, particularly when unexplained mucosal abnormalities are present. Endoscopists, pathologists, nephrologists, and hematologists must collaborate closely to ensure timely diagnosis and appropriate management of both localized and systemic manifestations of amyloidosis. Early recognition and intervention are critical in managing systemic amyloidosis, as they can significantly improve prognosis and reduce the risks of irreversible organ damage (26).

### Limitations and future perspectives

This case report underscores important clinical and diagnostic considerations; however, it is based on a single patient. The findings regarding NBI color patterns in gastrointestinal amyloidosis require validation through larger, multicenter studies to assess their diagnostic accuracy and reliability. Furthermore, the development of standardized endoscopic criteria for GIA would enhance the ability to detect this condition earlier and more reliably (27, 28). Looking ahead, the integration of artificial intelligence (AI) and machine learning into image-enhanced endoscopy could further refine the diagnostic process (29, 30). AI-powered systems, combined with advanced imaging modalities such as hyperspectral imaging and NBI, hold promise for improving diagnostic accuracy by analyzing fine-grained spectral differences in endoscopic images (31–33). These advancements could reduce diagnostic errors and facilitate the early detection of rare conditions such as GIA, ultimately improving clinical outcomes.

## Conclusion

Gastrointestinal amyloidosis is a rare and often underdiagnosed condition that presents significant diagnostic challenges (12). The combination of image-enhanced endoscopy (particularly NBI), targeted biopsies, and specialized staining techniques such as Congo red can improve early diagnosis, reduce misdiagnosis, and facilitate timely intervention (4, 34). Multispecialty collaboration is essential for the effective management of this systemic disease, and future research should focus on validating NBI as a diagnostic tool for GIA and exploring the role of AI in advancing gastrointestinal endoscopy.

### Patient perspective

From the patient’s point of view, the long interval between the first endoscopy and the final diagnosis of systemic amyloidosis was both confusing and anxiety-provoking. He initially believed that his gastric findings were minor and self-limited and did not expect them to be related to a systemic disease affecting the kidneys and heart. Looking back, he expressed some regret about not attending the recommended follow-up visit after the first gastroscopy but also relief that a definitive diagnosis was eventually established and treatment was started. He reported that understanding the cause of his symptoms and viewing the endoscopic and pathological images helped him to accept the diagnosis and to actively participate in treatment decisions. He hopes that sharing his experience will help clinicians and other patients recognize similar signs earlier and avoid delays in diagnosis.

## Data Availability

The original contributions presented in the study are included in the article/Supplementary material, further inquiries can be directed to the corresponding author.
