# Supplementary material for: Case Report: Image-enhanced endoscopic characteristics of gastric amyloidosis with narrow-band imaging comparison
Source: Front Med (Lausanne). 2026 Jan 29;13:1683757. doi: 10.3389/fmed.2026.1683757 (PMC12894309; doi:10.3389/fmed.2026.1683757)
Supplement: Supplementary file 1 [file Table_1.DOCX]

Laboratory tests showed a white blood cell count of 6.53 × 10⁹/L (reference: 3.5–9.5 × 10⁹/L), neutrophil count of 4.34 × 10⁹/L (reference: 1.8–6.3 × 10⁹/L), hemoglobin level of 115 g/L (reference: 130–175 g/L), and platelet count of 243 × 10⁹/L, indicating anemia. Urinalysis revealed 34 red blood cells/μL and 2+ proteinuria. The 24-hour urine protein quantification was 4106.17 mg/24 h (reference: <150 mg/24 h). Serum albumin was low at 27.5 g/L (reference: 40–55 g/L), creatinine was elevated at 118.8 μmol/L (reference: 59–104 μmol/L), and the estimated glomerular filtration rate (eGFR) was 55.8 mL/min (reference: >90 mL/min), indicating renal dysfunction. The erythrocyte sedimentation rate (ESR) was significantly elevated at 102 mm/h (reference: 0–15 mm/h), and CRP was elevated at 29.56 mg/L (reference: 0–6 mg/L), suggesting active inflammation. Urine specific proteins and light chain analysis revealed immunoglobulin G (IgG) at 37.4 mg/L (reference: <9.6 mg/L), transferrin at 76.8 mg/L (reference: <2.5 mg/L), microalbumin at 1460 mg/L (reference: <30 mg/L), α1-microglobulin at 20.4 mg/L (reference: <12 mg/L), λ-light chains at 26.4 mg/L (reference: <3.9 mg/L), and κ-light chains at 21.8 mg/L (reference: <7.1 mg/L). Serum immunofixation electrophoresis showed IgA positivity (+) and weak λ-chain positivity (±), with monoclonal IgA-λ detected. Urine immunofixation electrophoresis showed weak IgA positivity (±) and weak λ-chain positivity (±), with monoclonal IgA-λ detected. Stool routine, thyroid function, ANA, ANCA, and anti-glomerular basement membrane antibody tests were all negative.
